# Moist Wound Healing and Eutectic Mixture of Local Anesthetics Cream for Clean Buttonhole Cannulation: An Infection Reduction Strategy

**DOI:** 10.34067/KID.0000000864

**Published:** 2025-06-17

**Authors:** Kazuhiko Shibata, Shigeki Toma, Shigeru Nakai, Masumi Yamamoto, Kouichi Tamura

**Affiliations:** 1 Toshin Clinic, Yokohama, Japan; 2 Toma Clinic, Chatan, Japan; 3 Faculty of Clinical Engineering Technology, Fujita Health University School of Health Sciences, Toyoake, Japan; 4 Department of Medical Science and Cardiorenal Medicine, Yokohama City University Graduate School of Medicine, Yokohama, Japan

**Keywords:** hemodialysis access

## Abstract

**Key Points:**

- In buttonhole cannulation, where scabs are forcibly removed, the infection rate is higher than that in the rope ladder method.
- The clean buttonhole method enhances scab care by combining moist wound healing, thorough washing, and eutectic mixture of local anesthetics.
- The buttonhole technique allows patients to maintain healthy skin, except for the two puncture sites.

**Background:**

The buttonhole cannulation technique is recognized for its advantages in hemodialysis, including reduced cannulation errors, decreased pain, and prevention of hematoma formation. However, it has been criticized for possibly increasing the risk of local and systemic infections, including sepsis and death. Prior studies have shown the infection risks associated with the buttonhole technique compared with the rope ladder technique, but the role of scab removal methods at the puncture site has been overlooked. The clean buttonhole method was developed on the basis of softening and removing buttonhole scabs with liquid soap in home hemodialysis, incorporating moist wound-healing treatment, thorough washing, and eutectic mixture of local anesthetics cream to efficiently eliminate scabs.

**Methods:**

This observational study included patients undergoing maintenance hemodialysis at Toshin Clinic in Yokohama, Japan, from June 2020 to July 2024. Infection rates were documented in both the clean buttonhole and sharp needle groups. Statistical tests were used to compare the incidence of local infections between the groups.

**Results:**

Among 33,137 punctures performed using the clean buttonhole technique, only one localized infection was reported, characterized by mild redness without pain. By contrast, the sharp needle group experienced multiple infections with the rope ladder technique. The local infection rate was 0.030 cases per 1000 punctures in the clean buttonhole group compared with 0.025 cases per 1000 punctures in the sharp needle group, with no statistically significant difference observed (*P* = 0.86).

**Conclusions:**

The clean buttonhole method demonstrates a comparable infection risk profile with traditional sharp needle techniques. These findings indicate that this method may enhance patient safety and comfort in hemodialysis. Further research with larger, multicenter populations is needed to validate these results and assess broader applications in various clinical settings.

## Visual Abstract

**Figure:**
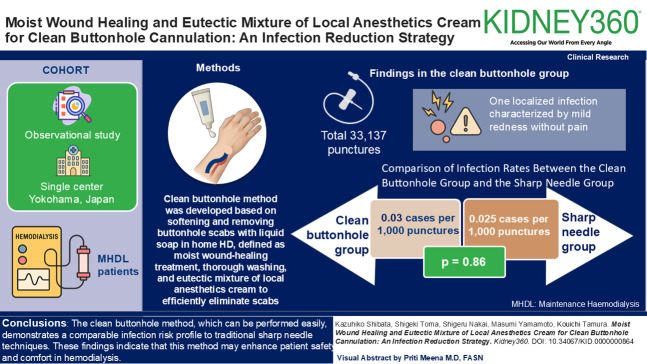

## Introduction

**Figure 1:**
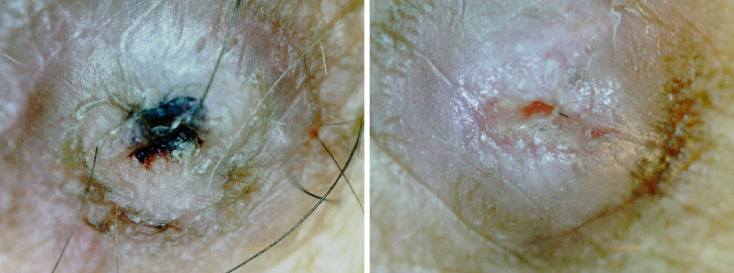
**Scab formation is inhibited by moist wound treatment immediately after hemodialysis for buttonhole.** Left: Before the initiation of moist wound healing treatment; scabs are observed. Right: After the initiation of moist wound-healing treatment; no scabs are present. Both images were taken before disinfection at the time of the visit.

The buttonhole cannulation technique has been reported to offer several advantages in hemodialysis, such as reduction in cannulation errors, decrease in pain, and prevention of hematoma formation.^1–6^ However, this technique is associated with an increased risk of local infection, systemic infection, and serious complications, such as sepsis or even death.^7–12^ Many studies have reported on the infection risks associated with arteriovenous fistula cannulation in hemodialysis, particularly comparing the buttonhole technique with the rope ladder technique to be sure. In several studies, it has been reported that thorough staff education on scab removal and disinfection has led to a reduction in infection rates that is comparable with the rope ladder method.^13^ However, one specific issue has been overlooked in these comparative studies evaluating infection rates between the buttonhole technique and other cannulation methods. In many of these studies, the scab that forms at the buttonhole site is often left to dry, harden, and adhere to the buttonhole entrance before the next hemodialysis. This scab must then be forcibly removed using sharp instruments, such as forceps, needles, or tweezers.^14^ This procedure not only causes pain to the patient but also risks damaging the skin at the buttonhole entrance during scab removal, creating a vicious cycle of more robust adhesion of the scab to the wound. From 2012 and over several years at the ERA-EDTA Congress, K. Shibata presented research showing that moist wound healing of buttonhole sites after hemodialysis reduced the size of scabs. As a result, the incidence of cannulation-related infections was effectively reduced to a level comparable with that of the rope ladder technique. In addition, puncture pain and elevated salivary amylase levels, a stress marker, were significantly lower in the buttonhole group.^15–17^ We also presented on moist wound healing for buttonhole entry sites at the 2012 Annual Meeting of the Japanese Society for Dialysis Therapy (Figure 1). However, in these trials, it is essential to note that any remaining scab fragment was carefully removed by our skilled nurses using instruments such as tweezers. This process is labor intensive and increases the staff workload compared with the rope ladder method, which only requires disinfection and cannulation. In addition, a barrier to the wider adoption of buttonhole punches may be the risk of infection and the perception that buttonhole care is cumbersome and difficult.

On the other hand, effective and easy methods for the removal of residual scab at the buttonhole site have been reported in the field of home hemodialysis. Stuart Mott, LNP, proposed a revolutionary method for removing scabs in patients on home hemodialysis.^18,19^ The method consists of applying liquid soap and gauze to the scab for 10 minutes before gently wiping it off. This approach allows for the removal of the entire scab without the use of sharp instruments such as tweezers, thereby avoiding the creation of new wounds around the buttonhole entry site. Although Mott's scab removal method is effective and does not require careful attention to avoid digging out scabs or special techniques, it is too time consuming for many hemodialysis units.

Despite its advantages, their study highlighted the high incidence of buttonhole infections among patients on in-facility hemodialysis. By contrast, home dialysis provides the opportunity for slower, more thorough scab removal, which has been shown to reduce infection rates.^19^ Thus, unfortunately, the excellent Mott scab removal method is not reaching patients in hospitals and dialysis centers. Given the time constraints in these facilities, there is a need for a simpler, less time-consuming method, an aspect that Mott's scab removal method does not address comfortably. Therefore, in this study, we evaluated a novel scab removal technique that combines the principles of moist wound healing with the Mott method specifically for buttonhole puncture sites. To make the procedure more feasible in busy hemodialysis centers, we developed an improved approach using eutectic mixture of local anesthetics (EMLA) cream to soften the scabs instead of a soap solution. While EMLA cream is specifically beneficial owing to its anesthetic effect, which helps reduce pain and improve patient compliance with the necessary steps, other lotions may be used as alternatives. This newly developed clean buttonhole method facilitates the safe and complete removal of visible scabs from buttonhole cannulation sites in a manner that is time efficient and comparable with standard hemodialysis cannulation approaches without requiring specialized skills. The clean buttonhole method made it easier to eliminate all the visible scabs. This may have helped to reduce the infection rate.

The aim of this research was to compare infection rates associated with the clean buttonhole method with those associated with the use of sharp needles, mainly rope ladder puncture, in hemodialysis.

## Methods

### Ethical Review and Study Period

This study received approval from the Kanagawa Medical Association Ethics Review Committee in March 2020 (no approval number was issued). The procedures followed in this study were in accordance with the ethical standards of the Declaration of Helsinki (1964, amended most recently in 2013), as developed by the World Medical Association. The participants of this study were patients who were undergoing maintenance hemodialysis at the Toshin Clinic (Yokohama, Japan) from June 2020 to July 2024 and provided written informed consent for participation. Patients who received arteriovenous grafts or long-term indwelling catheters were excluded from this study.

The cannulation method is described as follows.

#### Clean Buttonhole Method

**Figure 2:**
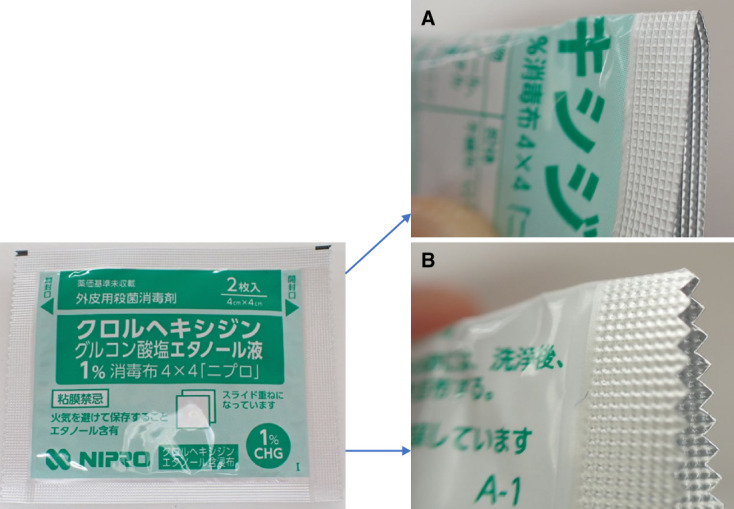
**Aluminum case for scab removal at the buttonhole entry site.** The aluminum pouch containing disinfectant cotton is folded in half, and the corner is used to hook onto the scab and remove it by gently pulling. As the edges of the pouch are not as hard as tweezers, skin damage is prevented. The flat edge (A) is suitable for easily removable scabs, whereas the jagged edge (B) is more effective for scabs that are firmly attached. After scab removal using this method, thorough disinfection of the area is performed to prevent infection.

The detailed procedure of the clean buttonhole method (Table 1) is as follows: First, the needle was removed, and hemostasis was achieved; subsequently, a bandage with a small amount of white petrolatum was used to cover the buttonhole cannulation site. This step was performed to reduce the size of the scab and promote epithelialization by providing a moist wound-healing method in the area of the buttonhole entry site. Once home, patients were instructed to remove the bandage, wash the area thoroughly, and then apply petroleum jelly to the site without covering it with a bandage. The petroleum jelly is a nonsterile white petrolatum formulation widely used in Japan as a moisturizing agent. Although the petroleum jelly is not sterilized, it is commonly and safely used in wound care owing to its protective and moisturizing properties. It is individually prescribed for each patient and reused exclusively by the same patient. Because patients were concerned about bleeding that may be caused by a nylon towel used to wash the buttonhole site, they preferred to gently clean the area with their palms using soap. However, they were encouraged to use commercially available nylon towels and soap to clean the buttonhole entry site until the scab was no longer visible. Despite their concerns, no bleeding occurred after gentle washing with nylon towels. Two hours before each hemodialysis session, EMLA cream was applied to the cannulation site and secured with a protective cover, which is an adhesive sheet made of cellophane (Sato Pharmaceutical, Tokyo, Japan) and included with the EMLA cream. Immediately before cannulation, the protective cover was removed, and the EMLA cream was wiped with sterile gauze and rubbed vigorously with cotton disinfectant to remove the crust. In most cases, this procedure made the scab nearly invisible. Any remaining scab was gently removed using the corners of a folded aluminum case containing disinfectant cotton packages while taking care to avoid skin damage (Figure 2). Disinfection was repeated before the puncture was performed by a doctor, nurse, or technician using 15- or 16-gauge cannulation needles (Supercath Clump Cath P painless needles; Medikit Co., Ltd., Tokyo, Japan), because almost all dialysis procedures performed in Japan use cannulas. We named this the clean buttonhole method, and the group of patients who received this method was referred to as the clean buttonhole group.

#### Sharp Needle Method

**Table 1:** Modified protocol for preparing the buttonhole cannulation site to maintain the clean buttonhole technique

Protocol
(1) After the hemodialysis session, cover the entry site with petroleum jelly to keep the site moist
(2) Scrub the puncture site at home with a nylon towel and soap and then apply petroleum jelly
(3) Two hours before hemodialysis puncture, apply EMLA cream and cover the site with a special cover
(4) Remove the cover and wipe the site with sterile gauze; scrub with 1% chlorhexidine alcohol monocotton. Usually, the buttonhole hole is clean and free of crusts and can be punctured
(5) If even the slightest crust is visible in the buttonhole, fold the aluminum bag of antiseptic single-use cotton in two and gently remove the crust with the corner. It can be punctured after redisinfection

EMLA, eutectic mixture of local anesthetics.

The rope ladder technique was primarily used for patients undergoing cannulation with sharp needles. In some patients, the area method or constant-site method was used. The group of patients undergoing cannulation with sharp needles (15- or 16-gauge needles; Supercath Clump Cath P needles, Medikit Co., Ltd.) was collectively referred to as the sharp needle group.

The disinfection method is described as follows: In both the clean buttonhole and sharp needle groups, the cannulation site was rubbed with individually packaged aluminum foil disinfecting cotton ten times before cannulation. The disinfectants used were as follows.

• Sharp needle group: 1% chlorhexidine cotton, alcohol-containing disinfecting cotton, and 10% povidone-iodine cotton.
• Clean buttonhole group: 1% chlorhexidine and 70% alcohol-containing disinfecting cotton, 10% povidone-iodine, and 1% chlorhexidine cotton.

### Indications for the Clean Buttonhole Method

The clean buttonhole method was applied to patients who met the following criteria: limited cannulation sites, cannulation pain, bulging deformities at the vascular access making the rope ladder technique difficult, young women and others who prefer to avoid vascular access deformities, and individuals who expressed a preference for this method. In cases involving arteriovenous grafts, the use of buttonhole puncture was prohibited by the ethics committee.

Before the study began in April 2020, two patients had already undergone the standard buttonhole technique and received guidance for the clean buttonhole method. Subsequently, we proactively transitioned from the sharp needle group to the clean buttonhole group. During the ethical review process, it was determined that the patients should not be compelled to continue buttonhole cannulation for data collection. Consequently, if patients in the clean buttonhole group had difficulty achieving smooth buttonhole cannulation, they were permitted to return to the sharp needle group. This flexibility led to some patients crossing over between the two groups.

## Results

The study protocol was approved by the ethics committee before the observational study, which involved clinically justified punctures. Owing to these ethical considerations, some patients who transitioned to the clean buttonhole group faced challenges in continuing with the buttonhole technique and subsequently reverted to the sharp needle group. Therefore, we presented the patient background and blood test data for both groups annually, comparing the number of infections with the total number of punctures. The local infection rate was defined as the proportion of punctures that resulted in local infections to the total number of punctures performed on targeted patients. The units for this measurement were expressed as cases per 1000 punctures.

Local infection was defined as the presence of any of the following signs at the puncture site: redness, purulent drainage, local warmth, or tenderness. The diagnosis of infection was made by the attending physician on the day of the observation. Cases in which there was redness without tenderness or where the appearance resembled contact dermatitis (such as that occurring at adhesive bandage sites) were excluded from both groups. Furthermore, during the entire observation period, no patients required hospitalization or died; thus, it was not possible to assess hospitalization bacteremia and mortality rates.

### Blood Test Parameters

Parameters such as dialysis quantity, protein catabolic rate, and Geriatric Nutritional Risk Index, dry weight, white blood cell count, and C-reactive protein were also evaluated.

### Statistical Analysis

Chi-squared test was used to evaluate the significance of the incidence of infections by comparing the frequencies of events between the groups. This statistical method was also applied to analyze patient background characteristics, including the prevalence of comorbidities and sex distribution. Conversely, Student's *t* test was used to assess continuous variables, such as age and laboratory test results. A significance level of <0.05 was established for all two-tailed tests.

### Patient Background

At the start of the observation period in June 2020, the clean buttonhole group consisted of ten patients (six male and four female), with an average age of 64.6±15.6 years and average dialysis duration of 137±127.3 months. The sharp needle group included 141 patients (95 male and 46 female), with an average age of 72.3±11.6 years and average dialysis duration of 133.0±105.7 months. As the transition to the buttonhole approach was encouraged, the number of patients in the clean buttonhole group gradually increased. Patients who transitioned from the clean buttonhole group to the sharp needle group continued to use constant-site punctures while they were away but did not experience any infections during that time before returning to the clean buttonhole group. Patient backgrounds for both groups are presented annually (Table 2). There were no statistically significant differences between the two groups in terms of age, gender ratio, dialysis history, or underlying diseases in any year. The dry weight in the clean buttonhole group was significantly higher in 2021; aside from this, no significant differences were observed between the two groups.

**Table 2:** Data and underlying conditions resulting in dialysis in patients participating at various time points in the clean buttonhole and sharp needle groups (June 2020–July 2024)^a^

	2020		*P* Value	2021		*P* Value	2022		*P* Value	2023		*P* Value	2024		*P* Value
	Buttonhole	Sharp	Student *t* Test	Buttonhole	Sharp	Student *t* Test	Buttonhole	Sharp	Student *t* Test	Buttonhole	Sharp	Student *t* Test	Buttonhole	Sharp	Student *t* Test
No. of patients	10	141		33	113		34	110		34	98		32	93	
Sex (male/female)	6/4	95/46	1^b^	7/26	73/40	0.12^b^	9/25	79/31	0.81^b^	11/23	75/23	0.84^b^	11/21	66/30	0.7^b^
Age (yr)	64.6±15.6	72.3±11.6	0.1	71.9±13.4	72.3±11.2	0.7	73.0±10.8	71.9±11.3	0.3	73.3±10.3	70.9±11.3	0.26	68.4±12.4	70.9±11.8	0.32
Dialysis history	137±127.3	133.0±105.7	0.4	79.2±252.2	130.0±109.1	0.1	114.1±112.3	85.6±92.2	0.9	101.9±95.7	105.7±104.0	0.81	103.0±94.0	104.6±104.7	0.93
Dry weight	57.8±17.5	59.2±15.4	0.78	66.2±15.6	60.7±14.9	0.01	62.2±12.8	61.4±16.2	0.8	62.6±15.1	61.2±16.5	0.66	66.9±16.1	62.1±16.3	0.16
Dialysis duration	4.1±0.4	4.1±0.4	0.92	4.08±0.29	4.06 ± 0.37	18.3392	4.04±0.33	4.08±0.36	0.33	4.10±0.27	4.1±0.35	0.61	4.10±0.3	4.09±0.28	0.89
Kt/V	16±0.3	1.5±0.2	0.71	1.49±0.13	1.51±0.19	0.36	1.47±0.16	1.49±0.20	0.42	1.53±0.15	1.50±0.13	0.21	1.52±0.15	1.52±0.16	0.97

	2020			2021			2022			2023			2024		
	Buttonhole	Sharp	Chi-Square Test	Buttonhole	Sharp	Chi-Square Test	Buttonhole	Sharp	Chi-Square Test	Buttonhole	Sharp	Chi-Square Test	Buttonhole	Sharp	Chi-Square Test
ANCA-associated nephritis													1		0.09
IgA nephropathy/Henoch–Schönlein purpura nephritis													1		0.09
Amyloidosis														1	0.56
Others	1	6	0.4				1	5	0.68	5	17	0.72	1	5	0.61
Lupus nephritis		3	0.64				1		0.07						
Malignant hypertension emergency		1						1	0.58				1	3	0.98
Renal and urinary tract tuberculosis								1	0.58						
Nephrosclerosis				2	5	0.7	2	19	0.1	16	33	0.16	4	12	0.95
Congenital renal and urinary tract abnormalities		1	0.64											1	0.56
Polycystic kidney disease		13	0.32	2	21	0.08	1	4	0.85		3	0.3		5	0.18
Diabetes mellitus	4	38	0.37	16	48	0.54	24	62		12	37	0.8	14	45	0.65
Unknown		12	0.32		1	0.59		4					1	4	0.77
Obstructive uropathy							1	2	0.69					1	0.56
Chronic GN	5	65	0.98	13	38	0.54	4	12	0.26	1	8	0.3	8	15	0.26
Chronic pyelonephritis		2	0.7										1		0.09
Drug-induced nephropathy														1	0.56
Total	10	141		33	113		34	110		34	98		32	93	

a *P* values < 0.05 according to chi-squared and Student *t* tests were considered significant.

b Evaluated by chi-squared test.

### Blood Test Data

In 2020, C-reactive protein levels were significantly higher in the clean buttonhole group than in the sharp needle group. In 2021, the white blood cell count was also significantly higher in the clean buttonhole group. However, the average values for both parameters remained within the normal range in both groups. No significant differences in other parameters were found after the introduction of 1% hexedine alcohol in 2022 (Table 3).

**Table 3:** Data on patients participating at various time points in the clean buttonhole and sharp needle groups^a^

	2020		*P* Value	2021		*P* Value	2022		*P* Value	2023		*P* Value	2024		*P* Value
	Buttonhole	Sharp		Buttonhole	Sharp		Buttonhole	Sharp		Buttonhole	Sharp		Buttonhole	Sharp	
CRP level (mg/L)	1.0±2.0	0.3±0.6	0.007	0.5±0.82	0.39±0.83	0.3	0.25±0.30	0.37±0.58	0.23	0.44±1.07	0.43±0.74	0.94	0.18±0.17	0.38±0.73	0.13
White blood cell count (cells/*μ*l)	6390±1674	6268±1732	0.71	6942±2168	6317±1708	0.02	6788±2132	6292±1735	0.13	6179±1842	6367±2039	0.64	6115±2199	6024±1711	0.74
nPCR (mg/kg body weight per day)	0.89±0.22	0.88±0.16	0.93	0.89±0.15	0.89±0.17	0.73	0.94±0.21	0.89±0.15	0.14	0.91±0.18	0.88±0.16	0.38	0.88±0.17	0.88±0.19	0.81
%CGR (%)	100.3±32.7	106.7±26.1	0.46	113.7±27.8	109.8±21.2	0.27	113.8±30.2	108.0±22.3	0.17	123.5±23.0	111.2±25.3	0.01	129.2±27.5	120.3±29.2	0.13
GNRI	96.5±8.0	959±5.8	0.74	98.9±4.4	96.6±5.7	0	99.4±4.85	96.9±6.04	0.02	96.5±5.5	96.2±6.4	0.8	94.0±6.0	91.4±6.63	0.06

CGR, creatinine generation rate; CRP, C-reactive protein; GNRI, Geriatric Nutritional Risk Index; nPCR, normalized protein catabolic rate.

a *P* values < 0.05 according to the Student *t* test were considered significant.

### Local Infection Rate

The total number of punctures was 33,137 for the clean buttonhole method and 162,074 for the sharp needle group (Table 4). The local infection rate for the clean buttonhole method was 0.030 cases per 1000 punctures, while the local infection rate for the sharp needle group was 0.025 cases per 1000 punctures. No significant difference was observed between these two methods (*P* = 0.86). One infection was counted in the clean buttonhole group; this case showed redness around the buttonhole but no spontaneous pain, tenderness, or pus drainage, and the redness decreased over 3 months. Although it was not possible to definitively establish whether this was caused by infection, the patient did not suffer any adverse effects. Blood cultures of the diagnosed infections were considered unnecessary by the attending physician, so they were not obtained. All infections in the sharp needle group occurred with the rope ladder method. The exact number of constant-site punctures is unknown; however, no infections have been recorded with this method. There were no patients in either group who required hospitalization for infection treatment, developed bacteremia, or died.

**Table 4:** Comparison of total puncture counts and infection occurrences over a 4-year observation period between the clean buttonhole and sharp needle groups^a^

	Total Punctures	Local Infection	Local Infection Incidence Rate (Cases per 1000 Punctures)	*P* Value	Bacteremia	Hospitalization	Death
Sharp needles	162,074	4	0.025	Control	0	0	0
Clean buttonhole	33,137	1	0.030	0.857	0	0	0

a *P* values were obtained from the chi-squared test, and those >0.05 were rejected.

## Discussion

The precise causes of infections that arise in conventional buttonhole punctures remain unclear. Most studies show that the buttonhole method causes infections, but there are a small number of recent meta-analyses, mainly from Asia, that show no significance.^3^ Previous literature has reported a high rate of infection when scabs are dried and removed with tools such as tweezers. This is likely to still be standard practice worldwide.^8,20,21^ By contrast, Stuart Mott and others reported that scab-softening techniques rarely resulted in infections, particularly in the Home Dialysis Central initiative, which recorded 54,094 cannulations across four countries without a single infection.^22^ This suggests that inadequate and incomplete removal of scabs may contribute to the increased infection rate. There are also reports indicating that infections can occur after scabs have been pushed into the skin.^19^

It can be postulated that bacteria invade the body using scabs as a medium. It is estimated that human skin harbors approximately 25,763 CFU/cm^2^ of bacteria.^23^ In addition, approximately 7.5 million *Staphylococcus aureus* cells are regarded as the minimum necessary to form a subcutaneous abscess.^24^ Assuming a 14 G puncture needle has a cross-sectional area of 3.46 mm^2^, the number of bacteria pushed into the skin during this process can be estimated at approximately 891 CFU. This number is drastically lower than the number of bacteria required to form a subcutaneous abscess. Conversely, if scabs are pushed into the subcutaneous tissue, they are warmed to body temperature and receive adequate moisture and nutrients, while simultaneously the scabs can protect the bacteria within from the immune system. *Staphylococcus aureus* grows rapidly in a rich medium with a doubling time of approximately 20 minutes. For many experiments, bacteria are grown to the exponential phase, which is characterized by a constant rate of cell division. Within two to three hours of incubation at 37°C, the culture typically reaches an approximate concentration of 1–5×10^7^ colony forming units.^25^ This bacterial load is sufficient to cause infection. Indeed, it has been reported that pushing scabs can lead to local infections.^19^

Our clean buttonhole technique visibly removed scabs completely in almost all cases, but there were some instances where we had to gently scoop out scabs with the edge of an aluminum case, avoiding the need for sharp instruments, such as tweezers. Stuart Mott's approach in home dialysis strictly avoided the use of picking instruments, and he reported that no infections occurred as a result. Our infection rate is also low, but higher than that reported by Mott, possibly because of his more rigorous approach allowing only wiping to remove scabs. However, considering that our method has been modified to be less time consuming and easier to perform at any dialysis center, this infection rate may be acceptable. The complete removal of visible scab at the puncture site with both techniques may have been a key factor in the lower infection rate compared with the conventional buttonhole technique.

The infection frequency in both groups can be considered sufficiently safe. This indicates that the risk of local infections associated with the clean buttonhole technique is at least comparable with the infection risk associated with the traditional sharp needle puncture method.

The clean buttonhole technique makes it easy to use in any hemodialysis facility by replacing a 10-minute waiting period with liquid soap using EMLA cream. The clean buttonhole method, which was simple and showed safety equivalent to the rope ladder method, is considered to be a viable alternative to the rope ladder method in many cases.

This study provides significant insights into the selection of puncture methods in clinical settings. The clean buttonhole method with moist treatment and EMLA cream demonstrated safety comparable with punctures performed with the sharp needle group.

However, there are several limitations to this study. First, as an observational study lacking patient randomization, there are inherent limitations in establishing causal relationships, and the assessment of infection rates relies on the subjective judgment of health care professionals. In addition, factors such as small sample size, all limitations inherent to study design, and the potential for selection bias further affect the findings. Furthermore, the study's participants were limited to a specific facility, necessitating caution in generalizing the results. The effect of moist treatment after needle withdrawal on infection rates also has yet to be established. Future research using different facilities and larger datasets is warranted.

The rope ladder technique is a highly effective method for long-term and stable punctures; however, it is often associated with significant pain and extensive skin trauma, leading many patients to experience discomfort. By contrast, the buttonhole method offers advantages such as reduced pain, lower puncture stress, and minimal skin damage or deformity in the arm. The results of this study indicate that the clean buttonhole group has a comparable risk with the sharp needle group, which primarily used the rope ladder technique. Our method has already been presented at various academic conferences and research meetings in Japan, gaining support and recognition as it continues to spread. We aspire to build on this momentum and hope to collaborate on expanding this research into larger studies with greater sample sizes to further validate our findings.

## Supplementary Material

## Data Availability

All data are included in the manuscript and/or supporting information.
