# Supplementary material for: Moist Wound Healing and Eutectic Mixture of Local Anesthetics Cream for Clean Buttonhole Cannulation: An Infection Reduction Strategy
Source: Kidney360. 2025 Jun 17;6(10):1762–70. doi: 10.34067/KID.0000000864 (PMC12778005; doi:10.34067/KID.0000000864)
Supplement: Supplementary file 1 [file kidney360-6-1762-s001.pdf]

ASN American Society of Nephrology
JASN Journal of the American Society of Nephrology
KIDNEY360 Accessing Our World From Every Angle
CJASN Clinical Journal of the American Society of Nephrology

ASN Journal Disclosure Form

As per ASN journal policy, I have disclosed any financial relationships or commitments I have held in the past 36 months as included below. I have listed my Current Employer below to indicate there is a relationship requiring disclosure. If no relationship exists, my Current Employer is not listed.

S. Nakai reports the following:
Employer: Medical Corporation Yuushin-kai, Daiko Medical Engineering Research Institute

I understand that the information above will be published within the journal article, if accepted, and that failure to comply and/or to accurately and completely report the potential financial conflicts of interest could lead to the following: 1) Prior to publication, article rejection, or 2) Post-publication, sanctions ranging from, but not limited to, issuing a correction, reporting the inaccurate information to the authors' institution, banning authors from submitting work to ASN journals for varying lengths of time, and/or retraction of the published work.

Name: Shigeru Nakai
Manuscript ID: K360-2025-000173R1
Manuscript Title: Moist wound healing and eutectic mixture of local anesthetics cream for clean buttonhole cannulation: An infection reduction strategy.
Date of Completion: April 24, 2025
Disclosure Updated Date: April 24, 2025

ASN American Society of Nephrology | JASN Journal of the American Society of Nephrology | KIDNEY360 Accessing Our World From Every Angle | CJASN Clinical Journal of the American Society of Nephrology

ASN Journal Disclosure Form

As per ASN journal policy, I have disclosed any financial relationships or commitments I have held in the past 36 months as included below. I have listed my Current Employer below to indicate there is a relationship requiring disclosure. If no relationship exists, my Current Employer is not listed.

K. Shibata reports the following:
Patents or Royalties: I have licensed patents to Medikit Co.

I understand that the information above will be published within the journal article, if accepted, and that failure to comply and/or to accurately and completely report the potential financial conflicts of interest could lead to the following: 1) Prior to publication, article rejection, or 2) Post-publication, sanctions ranging from, but not limited to, issuing a correction, reporting the inaccurate information to the authors' institution, banning authors from submitting work to ASN journals for varying lengths of time, and/or retraction of the published work.

Name: Kazuhiko Shibata
Manuscript ID: K360-2025-000173R1
Manuscript Title: Moist wound healing and eutectic mixture of local anesthetics cream for clean buttonhole cannulation: An infection reduction strategy.
Date of Completion: April 16, 2025
Disclosure Updated Date: April 16, 2025

ASN American Society of Nephrology | JASN Journal of the American Society of Nephrology | KIDNEY360 Accessing Our World From Every Angle | CJASN Clinical Journal of the American Society of Nephrology

ASN Journal Disclosure Form

As per ASN journal policy, I have disclosed any financial relationships or commitments I have held in the past 36 months as included below. I have listed my Current Employer below to indicate there is a relationship requiring disclosure. If no relationship exists, my Current Employer is not listed.

K. Tamura reports the following:
Employer: Yokohama City University Graduate School of Medicine; Research Funding: AstraZeneca, Bayer, Novartis, Chinook, Otsuka Medical Devices, Novo Nordisk, Terumo, Viatris, Kowa, Otsuka Pharmaceutical, Bayer, Mochida Pharmaceutical, Boehringer Ingelheim; Honoraria: AstraZeneca, Novartes, Bayer, Otsuka Pharmaceutical, Boehringer Ingelheim, Fuji Pharma, Kyowa Kirin, Ono Pharmaceutical, Sanwa Kagaku, Mochida Pharmaceutical, Kowa, Eli Lilly, Novo Nordisk; and Advisory or Leadership Role: Editorial Board: Hypertension Research, Kidney International Reports, Journal of Clinical Hypertension, Clinical and Experimental Nephrology, CEN Case Report, Circulation Journal, Journal of the Japan Medical Association and the Japanese Association of Medical Sciences (JMA Journal).

I understand that the information above will be published within the journal article, if accepted, and that failure to comply and/or to accurately and completely report the potential financial conflicts of interest could lead to the following: 1) Prior to publication, article rejection, or 2) Post-publication, sanctions ranging from, but not limited to, issuing a correction, reporting the inaccurate information to the authors' institution, banning authors from submitting work to ASN journals for varying lengths of time, and/or retraction of the published work.

Name: Kouichi Tamura
Manuscript ID: K360-2025-000173R1
Manuscript Title: Moist wound healing and eutectic mixture of local anesthetics cream for clean buttonhole cannulation: An infection reduction strategy
Date of Completion: April 21, 2025
Disclosure Updated Date: April 21, 2025

ASN American Society of Nephrology | JASN Journal of the American Society of Nephrology | KIDNEY360 Accessing Our World From Every Angle | CJASN Clinical Journal of the American Society of Nephrology

ASN Journal Disclosure Form

As per ASN journal policy, I have disclosed any financial relationships or commitments I have held in the past 36 months as included below. I have listed my Current Employer below to indicate there is a relationship requiring disclosure. If no relationship exists, my Current Employer is not listed.

S. Toma reports the following:
Employer: TOMA CLINIC

I understand that the information above will be published within the journal article, if accepted, and that failure to comply and/or to accurately and completely report the potential financial conflicts of interest could lead to the following: 1) Prior to publication, article rejection, or 2) Post-publication, sanctions ranging from, but not limited to, issuing a correction, reporting the inaccurate information to the authors' institution, banning authors from submitting work to ASN journals for varying lengths of time, and/or retraction of the published work.

Name: Shigeki Toma
Manuscript ID: K360-2025-000173R1
Manuscript Title: Moist wound healing and eutectic mixture of local anesthetics cream for clean buttonhole cannulation: An infection reduction strategy
Date of Completion: April 21, 2025
Disclosure Updated Date: April 21, 2025

ASN
American Society of Nephrology
JASN
Journal of the American Society of Nephrology
KIDNEY360
Accessing Our World From Every Angle
CJASN
Clinical Journal of the American Society of Nephrology

ASN Journal Disclosure Form

As per ASN journal policy, I have disclosed any financial relationships or commitments I have held in the past 36 months as included below. I have listed my Current Employer below to indicate there is a relationship requiring disclosure. If no relationship exists, my Current Employer is not listed.

M. Yamamoto reports the following:
Employer: Toshin Clinic

I understand that the information above will be published within the journal article, if accepted, and that failure to comply and/or to accurately and completely report the potential financial conflicts of interest could lead to the following: 1) Prior to publication, article rejection, or 2) Post-publication, sanctions ranging from, but not limited to, issuing a correction, reporting the inaccurate information to the authors' institution, banning authors from submitting work to ASN journals for varying lengths of time, and/or retraction of the published work.

Name: Masumi Yamamoto
Manuscript ID: K360-2025-000173R1
Manuscript Title: Moist wound healing and eutectic mixture of local anesthetics cream for clean buttonhole cannulation: An infection reduction strategy,
Date of Completion: April 30, 2025
Disclosure Updated Date: April 30, 2025
